# Complicity of α-synuclein oligomer and calcium dyshomeostasis in selective neuronal vulnerability in Lewy body disease

**DOI:** 10.1007/s12272-021-01334-6

**Published:** 2021-06-10

**Authors:** Kenji Yamamoto

**Affiliations:** Department of Neurology and Clinical Research Center, National Hospital Organization Utano National Hospital, 616-8255 Kyoto, Japan

**Keywords:** Parkinson’s disease, Dementia with Lewy body, α-Synuclein oligomers, Calcium dysregulation, Calcium binding protein, Selective neuronal degeneration

## Abstract

α-Synuclein oligomers and Ca^2+^ dyshomeostasis have been thoroughly investigated with respect to the pathogenesis of Lewy body disease (LBD). In LBD, α-synuclein oligomers exhibit a neuron-specific cytoplasmic distribution. Highly active neurons and neurons with a high Ca^2+^ burden are prone to damage in LBD. The neuronal vulnerability may be determined by transneuronal axonal transmission of the pathological processes; however, this hypothesis seems inconsistent with pathological findings that neurons anatomically connected to LBD-vulnerable neurons, such as neurons in the ventral tegmentum, are spared in LBD. This review focuses on and discusses the crucial roles played by α-synuclein oligomers and Ca^2+^ dyshomeostasis in early intraneural pathophysiology in LBD-vulnerable neurons. A challenging view is proposed on the synergy between retrograde transport of α-synuclein and vesicular Ca release, whereby neuronal vulnerability is propagated backward along repeatedly activated signaling pathway.

## Introduction

The pathological hallmarks of Lewy body disease (LBD), [also known as Parkinson’s disease (PD) and dementia with Lewy bodies] are Lewy bodies and Lewy neurites, which are composed of insoluble α-synuclein fibrils (Irvine et al. 2008). The LBD pathological process targets specific subcortical and cortical neurons in addition to dopaminergic neurons in the substantia nigra pars compacta (SNc) (Braak et al. 2003). In the peripheral nervous system, LBD pathology mainly appears in the autonomic nervous system (Braak et al. 2003; Surmeier and Sulzer 2013; Kalia and Lang 2015). In the central nervous system, lesions initially occur in the dorsal motor nucleus of the vagus nerve (DMV) and the anterior olfactory nucleus (Braak et al. 2003; Surmeier and Sulzer 2013; Kalia and Lang 2015). In the brainstem, Lewy pathology and cell loss have been observed in the region of the DMV, the medullary reticular formation, the raphe nuclei, the locus coeruleus, the pedunculopontine nuclei, and the SNc (Braak et al. 2003; Surmeier and Sulzer 2013; Kalia and Lang 2015). The Lewy pathology in the brainstem and the anterior olfactory nucleus expands into related areas such as the nucleus basalis of Meynert, the amygdala, and the cerebral cortex (Braak et al. 2003; Surmeier and Sulzer 2013; Kalia and Lang 2015). This selective neuronal involvement is the background not only of classical parkinsonian motor symptoms, but also of heterogeneous non-motor features, including autonomic dysfunction, olfactory dysfunction, sleep disorders, psychiatric symptoms, and cognitive impairment, all of which lower the quality of life in LBD patients (Chaudhuri et al. 2006). In contrast, neurons in the ventral tegmental area (VTA) and globus pallidus, striatal cholinergic interneurons, and cerebellar Purkinje neurons, which are anatomically connected to PD-vulnerable neurons, are not involved in PD (Braak et al. 2003; Surmeier and Sulzer 2013). Given that selective neuronal vulnerability in slowly progressing LBD pathology is incompatible with the simple idea of transneuronal retrograde axonal spreading of LBD pathology along anatomical connectivities (Del Tredici and Braak 2020), what is the basis of selective neuronal fragility in LBD?

This review focuses on two key features of neurons in the LBD brain that contribute to the pathophysiology of LBD-vulnerable neurons: α-synuclein oligomers and calcium dyshomeostasis. Also discussed is the recent finding that α-synuclein oligomers trigger an aberrant form of robust-firing-induced calcium release from the endoplasmic reticulum (ER), which is mediated by direct binding with calcium binding protein 1 (CaBP1), a neuron-specific member of the calmodulin superfamily. This binding disrupts the normal Ca^2+^- and IP_3_-dependent regulation of inositol 1, 4, 5-trisphosphate (IP_3_) receptors (IP_3_Rs) (Yamamoto et al. 2019). The present review proposes that this signaling-path-specific calcium dysregulation uniquely contributes to early oligomeric α-synuclein-mediated pathophysiology in LBD-vulnerable neurons.

### α-Synuclein oligomers: a pivotal factor in LBD pathogenesis

α-Synuclein is highly expressed in the brain and is enriched at presynaptic terminals. It augments assembly of the SNARE machinery, and plays a role in neurotransmitter release and protection of nerve terminals against injury (Kalia et al. 2013). The main physiological form of α-synuclein in the brain appears to be an unfolded monomer, but it also forms soluble oligomeric species that are toxic to neurons (Irvine et al. 2008; Dehay et al. 2015; Ingelsson 2016). The presence of α-synuclein oligomers has been demonstrated not only with recombinant proteins or in cell culture and in vivo models, but also in post-mortem brain tissue from LBD patients (Cappai et al. 2005; Yamakawa et al. 2010; Sharon et al. 2003; Paleologou et al. 2009). Proximity ligation assays of LBD brain tissue can detect α-synuclein oligomers, which can go undetected by conventional immunohistochemistry using anti-α-synuclein antibodies (Roberts et al. 2015). α-Synuclein oligomers can be observed not only in the neuropil, but also in the cytosol of LBD-vulnerable neurons, which can precede the development of classical PD lesions, such as pale bodies or Lewy bodies. (Roberts et al. 2015).

**Fig. 1:**
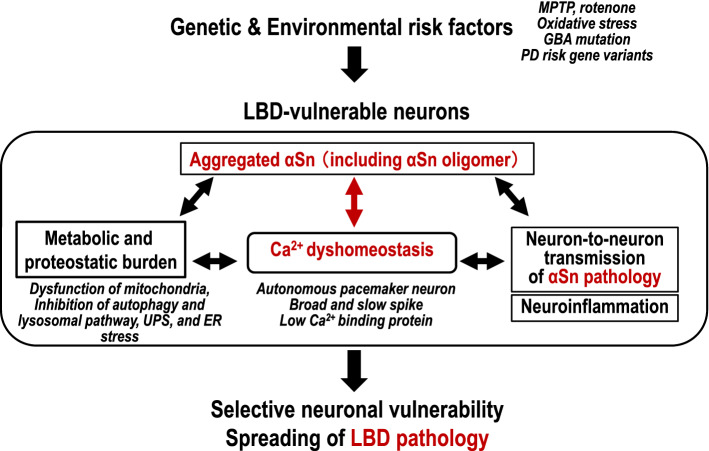
α-Synuclein oligomers and intraneural calcium dyshomeostasis play key roles in the pathophysiological mechanism of LBD. Genetic and environmental risk factors promote LBD pathology, including the production of α-synuclein (αSn) oligomers and the disturbance of Ca^2+^ homeostasis in LBD-vulnerable neurons, mutually strengthening the metabolic and proteostatic burden, and increasing the neuron-to-neuron propagation of αSn pathology and neuroinflammation. These pathophysiological alterations cause selective neuronal vulnerability and the spreading of LBD pathology. The metabolic and proteostatic burden includes mitochondrial dysfunction, the autophagy and lysosomal pathway and the ubiquitin proteasome system (UPS), and ER stress. LBD-vulnerable neurons share the features underlying Ca^2+^ dyshomeostasis, such as being autonomous pacemaker neurons, having broad and slow spikes, and low levels of Ca^2+^ binding protein. Note that neurons that do not have such Ca^2+^-related properties, such as neocortical neurons, are also prone to damage in LBD patients with cognitive decline

In sporadic PD, genetic risk factors, such as mutations in the glucocerebrosidase gene (*GBA*) (Oeda et al. 2015; Schapira 2015), and susceptibility variants in *SNCA*, *MAPT*, *LRRK2*, *PARK16*, and *BST1* have been identified by genome-wide association studies (Satake et al. 2009; Simón-Sánchez et al. 2009). The aggregation of α-synuclein is upregulated in variants of α-synuclein (Irvine et al. 2008) and LRRK2 (Aasly et al. 2014). α-Synuclein BAC transgenic mice show oligomeric forms of α-synuclein in the regions that are specifically affected in LBD, including the olfactory bulb, cerebral cortex, striatum and SNc, and exhibit non-motor symptoms, such as rapid eye movement sleep behavior disorder-like behavior and hyposmia (Taguchi et al. 2020). Environmental risk factors, including exposure to MPTP and rotenone, or oxidative stress, also cause LBD pathology (Irvine et al. 2008), while exposure to metals and pesticides can increase α-synuclein aggregation (Uversky et al. 2002; Irvine et al. 2008). α-Synuclein oligomer toxicity occurs via several intracellular mechanisms, including mitochondrial dysfunction, ER stress, impaired autophagy-lysosomal pathways (Dehay et al. 2015; Ingelsson 2016), and neuroinflammation (Kim et al. 2013; Rocha et al. 2018). Furthermore, oligomeric and fibril species of α-synuclein can be transferred between neurons, thereby propagating α-synuclein pathologies (Hansen et al. 2011; Kim et al. 2019). This wide array of studies indicates that the α-synuclein oligomer is a key toxic molecule of LBD-vulnerable neurons before Lewy bodies are formed and neuronal loss occurs (Fig. 1).

### Dysregulated Ca^2+^ homeostasis: a key feature of selective neuronal fragility in LBD

Dysregulation of Ca^2+^ homeostasis is a core pathological mechanism in LBD. It triggers the formation of α-synuclein oligomers, mitochondrial and ER stress, and inhibition of autophagy and lysosomal pathways, thereby leading to neurodegeneration (Surmeier and Sulzer 2013). In SNc neurons of aging mice, Ca^2+^ entry via CaV1.3, an L-type voltage-dependent Ca^2+^ channel (VDCC) during autonomous action potentials causes oxidative stress and cell damage (Chan et al. 2007; Surmeier and Sulzer 2013). Epidemiological studies indicate that L-type VDCC (L-VDCC) blockers diminish the risk of PD (Ritz et al. 2010; Ascherio and Schwarzschild 2016). Moreover, soluble oligomeric α-synuclein induces Ca^2+^ influx and seeding (Danzer et al. 2007), and promotes Ca^2+^-induced mitochondrial dysfunction (Luth et al. 2014). Taken together, these observations raise the possibility that the close relationship between α-synuclein oligomers and the dysregulation of intracellular Ca^2+^ handling by intraneural organelles may play a central role in LBD pathogenesis.

In general, LBD-vulnerable neurons, such as neurons in the SNc, the locus coeruleus, the raphe nuclei and the nucleus basalis of Meynert, have a common physiological phenotype; an autonomous pacemaker, broad and slow spiking, and low levels of Ca^2+^-binding protein expression (Surmeier and Sulzer 2013). These physiological properties lead to increased cytosolic Ca^2+^ and augment the metabolic burden in these neurons that is critical for selective neuronal degeneration (Surmeier and Sulzer 2013). Of note, the neurons in the VTA, which are LBD-resistant, have high neuronal activities as autonomous pacemakers, but express a high level of Ca^2+^-binding protein, indicating the importance of not only activity-dependent, but also signaling-path-specific Ca^2+^ burden for the selective neuronal vulnerability in LBD (Surmeier and Sulzer 2013). Isradipine, a dihydropyridine calcium-channel (CaV1.3) blocker approved for the treatment of hypertension, is neuroprotective in animal models of PD (Chan et al. 2007; Guzman et al. 2010; Ilijic et al. 2011). This neuroprotective effect is mediated by inhibition of plasma membrane L-type calcium channels, which trigger mitochondrial oxidant stress and turnover (Guzman et al. 2010; Ilijic et al. 2011). These studies prompted a clinical trial evaluating the efficacy of isradipine to slow the clinical progression of PD in previously untreated early-stage PD patients. However, this trial indicated no significant effect (The Parkinson Study Group STEADY-PD III Investigators 2020). Attention has, therefore, been directed to other Ca^2+^ channel-dependent mechanisms of activity-dependent Ca^2+^ dyshomeostasis and signaling-path-specific selective fragility of neurons in LBD brains.

Lewy pathology is neuron-specific in LBD, suggesting a crucial role of intraneural α-synuclein in LBD pathology that is distinct from the cytoplasmic α-synuclein inclusions of glial cells in multiple system atrophy (Jucker and Walker 2013; Del Tredici and Braak 2020). Lewy pathology appears in neocortical pyramidal neurons, which do not have properties related to Ca^2+^ dyshomeostasis, such as being autonomous pacemaker cells, exhibiting broad and slow spikes and low levels of Ca^2+^ binding protein (Yamamoto et al. 2019). This pathology contributes to dementia in LBD, the second major cause of degenerative dementia (Braak et al. 2003; Chaudhuri et al. 2006). An unsolved question is, therefore, how intraneural α-synuclein oligomers pathologically can alter neuronal activity and intracellular Ca^2+^ dynamics in neocortical neurons. By using intracellular injection of bioactive molecules or proteins such as inositol trisphosphate (IP_3_), homer1a and amyloid-β through a patch pipette, how Ca^2+^ and K^+^ channels are involved in the regulation or pathophysiological change of neocortical pyramidal cell excitability and Ca^2+^ dynamics are demonstrated (Sakagami et al. 2005; Yamamoto et al. 2000, 2002a, 2002b, 2005, 2011). Interestingly, the results obtained using these methods correspond to those observed in neurons with physiologically produced IP_3_ or homer1a proteins in the cytoplasm, or in neurons of 3xTg Alzheimer’s disease model mice (Cui et al. 2007; Nakamura et al. 1999; Sakagami et al. 2005; Stutzmann et al. 2003; Yamamoto et al. 2000, 2002a, 2002b, 2005, 2011). The same methodology was used to introduce α-synuclein protein into pyramidal neurons in cortical slices from mice to investigate the effects and mechanisms of intracellular α-synuclein oligomers on neuronal excitability and Ca^2+^ dynamics, as described below (Yamamoto et al. 2019).

### Aberrant activity-dependent calcium release from IP_3_ receptors in central neurons caused by the association of α-synuclein oligomers with CABP1

**Fig. 2:**
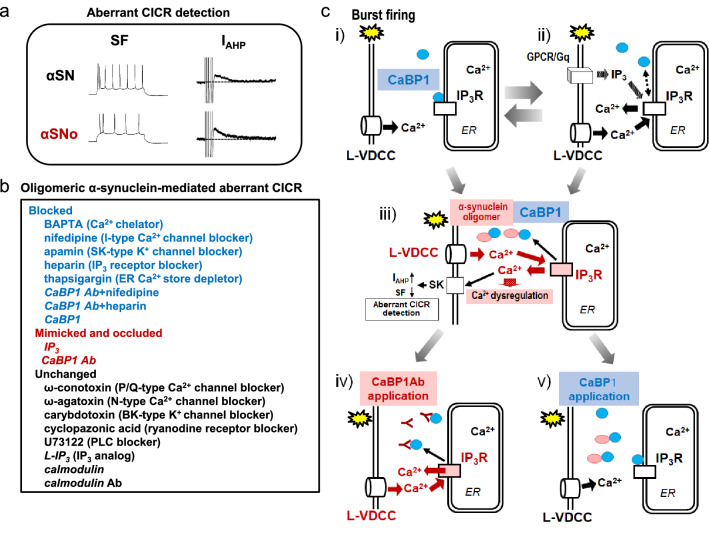
The electrophysiological detection and the mechanism of oligomeric α-synuclein-mediated aberrant Ca^2+^-induced Ca^2+^ release (CICR). **a** Aberrant CICR was detected by a decrease in spike frequency (SK) and an enhancement of I_AHP_ (afterhyperpolarization current) in αSNo (including higher-order α-synuclein oligomers)-applied neurons compared with αSN (free from higher-order α-synuclein oligomers)-applied neurons. After filtering to remove α-synuclein fibrils, αSNo and αSN solutions were used as pipette solutions for current clamp and voltage clamp recordings (Yamamoto et al. 2019). **b** The mechanism of oligomeric α-synuclein-mediated aberrant CICR was examined using drugs to modulate the channels and receptors that control intraneural Ca^2+^ dynamics, and biomolecules or antibodies that regulate these channels and receptors (Yamamoto et al. 2019). The aberrant CICR is blocked by the extracellular application (non-italic text) of blockers of L-VDCC, SK, and IP_3_R, a Ca^2+^ chelator, and an ER store depletor, and by the intracellular application (*italic text*) of calcium binding protein 1 (CaBP1), which is a neuron-specific regulator of IP_3_R gating and directly binds higher order α-synuclein oligomers (Yamamoto et al. 2019). The intracellular application of IP_3_ and CaBP1 antibodies (Ab) both occlude the effect of αSNo. **c** A scheme illustrating the aberrant CICR during burst firing (from Yamamoto et al. 2019 with partial modification). (i) Endogenous CaBP1 (blue circle) binds IP_3_Rs and maintains Ca^2+^-dependent inactivation of IP_3_Rs (white rectangle). (ii) IP_3_ elevation controlled by finely tuned neurotransmission or neuromodulation is required for physiological CICR from IP_3_Rs. GPCR/Gq: G-protein-coupled receptor/Gq protein. (iii) Intraneural α-synuclein oligomer (red ellipse) binds to endogenous CaBP1 and allows aberrant CICR from IP_3_Rs (red rectangle) independent of IP_3_ turnover, which enhances Ca^2+^ dysregulation. (iv) CaBP1 Ab binds endogenous CaBP1, prevents CaBP1 regulation of IP_3_Rs and triggers aberrant CICR in a similar manner to the α-synuclein oligomer. (v) CaBP1 binds α-synuclein oligomer and blocks oligomeric α-synuclein-mediated aberrant CICR

To clarify pathophysiological changes in neuronal activity regulating intraneural Ca^2+^ induced by α-synuclein oligomers, whole-cell recordings were obtained from pyramidal neurons located in slices of the mouse frontal cortex (Yamamoto et al. 2019). Immunoblotting analysis using anti-α-synuclein antibodies revealed that the recombinant α-synuclein incubated with dopamine at 37 °C for 3 days produced higher-order oligomers (αSNo) and fibrils, while the recombinant α-synuclein incubated without dopamine for 3 days was free of higher-order oligomers (αSN) (Yamamoto et al. 2019). After filtering to remove α-synuclein fibrils, αSNo and αSN solutions were used as pipette solutions, and the properties of action potentials in current clamp mode and the current charges during spike afterhyperpolarization (AHP) in voltage clamp mode were examined (Yamamoto et al. 2019). This examination revealed that intracellular application of αSNo significantly reduced spike frequency during current injection, elongated the duration of spike AHP, and enlarged AHP current charge compared with αSN (Yamamoto et al. 2019; Fig. 2). This αSNo-mediated alteration was triggered by spike-induced Ca^2+^ release from IP_3_Rs functionally coupled with L-VDCC and small conductance Ca^2+^ activated K^+^ (SK) channels under the application of blockers for the channels or receptors responsible for intraneural Ca^2+^ dynamics (Yamamoto et al. 2019; Fig. 2). This Ca^2+^-dependent functional triad consisting of L-VDCCs, IP_3_Rs and SK channels is well established and is linked to spike-triggered Ca^2+^ inflow and Ca^2+^ release (Ca^2+^-induced Ca^2+^ release; CICR) from IP_3_Rs in neurons of the neocortex and amygdala, and contributes to the regulation of neuronal excitability and synaptic plasticity Yamamoto et al. 2000, 2002a, b, Faber et al. 2010, Power and Sah 2005, 2008; Yamada et al. 2004; Fig. 2c, iii). In contrast with previous reports that emphasized how the physiological upregulation of IP_3_ turnover is finely tuned by synaptic stimulation or neuromodulation and is necessary for spike-induced or IP_3_-induced Ca^2+^ release from IP_3_Rs in central neurons (Cui et al. 2007; Faber et al. 2010, Nakamura et al. 1999; Power and Sah 2005, 2008; Stutzmann et al. 2003; Yamada et al. 2004; Yamamoto et al. 2000, 2002a, 2002b; Fig. 2c, ii), the oligomeric α-synuclein-mediated CICR from IP_3_Rs presented here was independent of increased IP_3_ production, because the phospholipase C (PLC) blocker, U73122, failed to inhibit it (Yamamoto et al. 2019; Fig. 2b). Such an unusual mode of CICR provoked by highly frequent neuronal activity, independent of IP_3_ turnover, does not usually occur in central neurons because the regulation of IP_3_R gating exhibits bell-shaped dependence on somatic Ca^2+^ concentration (Bezprozvanny et al. 1991). This mode of CICR can therefore be regarded as pathological, forcing an excess Ca^2+^ burden on neurons, surpassing a negative feedback regulation of spike firing (Fig. 2c, iii). Accordingly, via this channel coupling, α-synuclein oligomers provoke the aberrant CICR from IP_3_Rs, which is triggered by Ca^2+^ influx via L-VDCCs during repetitive firing, followed by elongation of SK channel opening (the prolongation of I_AHP_) and decreased spike frequency (Yamamoto et al. 2019). Consequently, in neocortical pyramidal neurons, the occurrence of this aberrant mode of CICR can be detected by examining the elevation of I_AHP_ charge and the reduction in spike frequency (Fig. 2c i, iii; Yamamoto et al. 2019).

IP_3_R has two separate binding sites for Ca^2+^ and IP_3_, which are allosterically regulated by the two ligands. The binding of one ligand facilitates additional binding of the other (Berridge 1998; Verkhratsky 2005); therefore, IP_3_R responds to the increase in neuronal cytosolic Ca^2+^ and IP_3_, opens, and releases Ca^2+^ from the ER in an activity-dependent manner (Yamamoto et al. 2000, 2002a, b; Yamada et al. 2004, Nakamura et al. 1999, Larkum et al. 2003). The PLC blocker, U73122, failed to block Ca^2+^ release but intracellular application of IP_3_ occluded the effect of αSNo; therefore, α-synuclein oligomers modulate IP_3_R gating and mediate the aberrant form of CICR from IP_3_Rs during repetitive spikes, without enhancing Ca^2+^ influx or IP_3_ production in neocortical neurons (Yamamoto et al. 2019; Fig. 2c, iii).

The gating of IP_3_R is not only regulated by IP_3_ or Ca^2+^ binding, but also by various proteins that interact with IP_3_R (Choe and Ehrlich 2006; Foskett et al. 2007); therefore, αSNo could be linked to a protein that directly binds IP_3_R and regulates IP_3_R gating in central neurons. Among the binding partners of IP_3_R, Ca^2+^-binding protein 1 (CaBP1) is the most promising candidate, because CaBP1 is (1) a Ca^2+^-binding protein expressed in the cytosol of rodent and human central neurons (Laube et al. 2002; Bernstein et al. 2003; Kim et al. 2014), (2) a protein that preferentially interacts with oligomeric α-synuclein (Betzer et al. 2015), and (3) a binding partner and negative regulator of IP_3_Rs under high intraneural Ca^2+^ concentration by means of Ca^2+^-dependent inactivation (Haynes et al. 2004; Kasri et al. 2004; Li et al. 2013). CaBP1 is a neuronal Ca^2+^-binding protein splice variant, a sub-branch of the calmodulin superfamily, which includes Ca^2+^-sensor proteins that regulate various Ca^2+^ channel targets (Yang et al. 2002; Haeseleer et al. 2000). CaPB1 has four EF-hand Ca^2+^-binding motifs, and can bind and regulate IP_3_R under high intraneural Ca^2+^ concentration (Haynes et al. 2004; Kasri et al. 2004; Li et al. 2013). Once αSNo binds to CaBP1 and pulls it away from IP_3_R, thus disrupting Ca^2+^-dependent regulation of IP3R, an aberrant CICR from IP_3_R can occur without enhancing Ca^2+^ influx or cytosolic IP_3_ concentration.

The effects of intracellular application of a CaBP1 antibody (Ab) and CaBP1 on αSNo-mediated change were compatible with this hypothesis. CaBP1 Ab occluded the effect of αSNo, which was blocked by nifedipine and heparin, indicating that αSNo-mediated capture of CaBP1 is necessary and sufficient for the aberrant CICR from IP_3_R (Yamamoto et al. 2019; Fig. 2c, iv, v). By contrast, calmodulin, which is another binding partner and regulator of IP_3_R (Yang et al. 2002; Taylor and Tovey 2010) and directly binds α-synuclein (Lee et al. 2002; Martinez et al. 2003), and a calmodulin antibody, failed to counteract αSNo-mediated action (Yamamoto et al. 2019; Fig. 2b). These electrophysiological observations were further supported by immunoprecipitation experiments that demonstrated the direct binding of higher-order α-synuclein oligomers larger than 100 kDa with CaBP1 (Yamamoto et al. 2019). Together, these electrophysiological findings demonstrate that the target of α-synuclein oligomers was CaBP1, and confirmed the direct association of α-synuclein oligomers greater than 100 kDa with CaBP1, resulting in aberrant CICR from IP_3_R (Yamamoto et al. 2019). These results are consistent with previous studies showing that transgenic α-synuclein mice exhibit augmented long-lasting Ca^2+^ transients in response to repetitive stimulation *in vivo* (Reznichenko et al. 2012), and that neocortical pyramidal cell excitability is reduced by injecting α-synuclein oligomers (Kaufmann et al. 2016), but emphasize that α-synuclein oligomers cause this activity-dependent signaling-path-specific CICR surpassing the regulation of spike firing.

IP_3_ and CaBP1 have opposing effects on the IP_3_R channel under high intracellular Ca^2+^ concentrations (Haynes et al. 2004; Karsi et al. 2004; Li et al. 2013). IP_3_ obstructs the inter-subunit interaction of IP_3_R and encourages IP_3_R channel opening (Choe and Ehrlich 2006; Li et al. 2013). By contrast, CaBP1 binds IP_3_R and prevents the inter-subunit interaction of IP_3_R when the somatic concentration of IP_3_ is low, thereby hindering IP_3_R channel opening in a Ca^2+^-dependent manner Choe and Ehrlich 2006; Li et al. 2013; Fig. 2c i). These mechanisms clarify why IP_3_ and CaBP1 Ab occlude and heparin and CaBP1 inhibit the action of αSNo (Fig. 2c, ii, iv, v); oligomeric α-synuclein-mediated removal of CaBP1-mediated regulation of IP_3_R, but not the Ca^2+^ buffering effect of CaBP1, is essential for aberrant CICR from IP_3_R (Fig. 2c, iii).

### A potential mechanism of oligomeric α-synuclein-mediated calcium dysregulation in early pathology of LBD-vulnerable neurons

IP_3_R is localized on ER membrane connected with mitochondria, known as the mitochondria-associated ER membrane, and increased Ca^2+^ transfer from the ER via IP_3_Rs to mitochondria inhibits autophagy (Szabadkai et al., 2008; Decuypere et al. 2011). Chronic aberrant CICR from the IP_3_R in the presence of intraneural α-synuclein oligomers may increase the risk of activity-dependent mitochondrial stress through the IP_3_R-mitochondrial connection, and may lead to neuronal fragility in oligomeric α-synuclein-bearing neurons. This notion is supported by previous studies indicating that α-synuclein can alter mitochondrial Ca^2+^ homeostasis by enhancing ER-mitochondria interactions (Cali et al. 2012), and that α-synuclein can be localized to the mitochondria-associated ER membrane (Guardia-Laguarta et al. 2014).

**Fig. 3:**
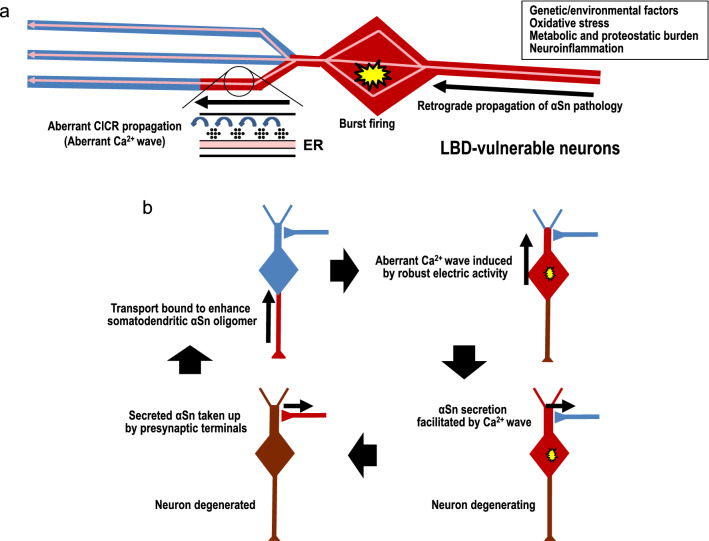
A potential mechanism of oligomeric α-synuclein-mediated calcium dyshomeostasis promoting early pathology in LBD-vulnerable neurons. **a** In LBD-vulnerable neurons, various LBD risk factors trigger a dying-back pathology that is initiated at synaptic terminals and progresses along the axon with oligomeric α-synuclein (αSn) appearing in cell bodies. During burst firing, the oligomeric αSn-mediated CICR from IP_3_Rs propagates as an aberrant Ca^2+^ wave via IP_3_Rs and ryanodine receptors along the somatodendritic portion of the ER (Ca^2+^: black dot). **b** A proposed retrograde bottom-up model of αSn propagation reinforced by the aberrant Ca^2+^ wave in LBD-vulnerable neurons. Retrograde propagation of αSn pathology (upper left) brings somatodendritic αSn oligomers, which cause chronic aberrant CICR propagation (aberrant Ca^2+^ wave) during burst firing (upper right). This Ca^2+^ dysregulation in turn promotes the Ca^2+^ burden, the secretion of αSn from dendrites (lower right), and transmission of αSn pathology to nearby synaptic terminals (lower left). Together with neuronal degeneration of LBD-vulnerable neurons, the retrograde propagation of αSn pathology in presynaptic LBD-vulnerable neurons is initiated (lower left, upper right). This mechanism specific to repeatedly activated signaling path can account for the propagation of LBD pathology

The ER is a continuous “neuron-within-neuron” network that extends to all parts of the neuron, including the spines, cell soma, and synaptic endings, and that supports regional and long-distance Ca^2+^ homeostasis (Berridge 1998; Öztürk et al. 2020). Ca^2+^ signals can transmit through the cytosol by CICR from the ER, and induce regional or global communication within the cell, at a pace that is slower than action potential propagation in the plasma membrane. CICR can be mediated by IP_3_Rs or ryanodine receptors and can be augmented by elevated cytosolic Ca^2+^ (Berridge 1998; Öztürk et al. 2020). The CICR from IP_3_Rs propagates as a Ca^2+^ wave along the ER via IP_3_Rs and ryanodine receptors throughout the somatodendritic portion and the nucleus (Power and Sah 2002; Watanabe et al. 2006; Ross 2012). This normal Ca^2+^ wave propagating from dendrite to soma and nucleus, is initiated only when strong synaptic stimulation or synaptic stimulation concurrent with repetitive spikes occurs (Power and Sah 2002; Watanabe et al. 2006; Ross 2012). This is mainly dependent on IP_3_R which is distributed in the soma and dendrites, thereby regulating neuronal excitability and synaptic plasticity (Power and Sah 2002; Watanabe et al. 2006; Ross 2012). In contrast, it is noteworthy that repetitive spikes alone, independent of synaptic input, are enough to cause the aberrant CICR from the ER via IP_3_Rs in the presence of α-synuclein oligomers (Yamamoto et al. 2019; Fig. 2c, iii). Subsequent to backpropagating action potentials from the soma to dendrites (Waters et al. 2005), this aberrant CICR can cause the chronic aberrant Ca^2+^ wave to be propagated along the soma-dendritic region (Yamamoto et al. 2019; Fig. 3). A sporadic PD risk gene, *BST1*, encodes cyclic ADP-ribose hydrolase 2, which synthesizes cyclic ADP-ribose, a ryanodine receptor agonist (Satake et al. 2009; Saad et al. 2011). *BST1* variants can disturb normal channel function of the ryanodine receptor, another Ca^2+^ release channel in the ER, and can enhance the aberrant CICR via IP_3_Rs to promote propagation of dysregulated Ca^2+^ waves (Yamamoto et al. 2019; Fig. 3). Chronic occurrence of this aberrant CICR and Ca^2+^ wave may increase the risk of distinct activity-dependent Ca^2+^ dyshomeostasis and may lead to pathological intraneural spreading and neuronal fragility specific to repeatedly activated signaling path in oligomeric α-synuclein-bearing neurons, although this remains to be studied. Considering that oligomeric α-synuclein accumulates first at presynaptic terminals in LBD (Bridi et al. 2018), and that the pathological change of LBD-vulnerable neurons is a dying back-phenomenon, which initiates at synaptic terminals and progresses along the axon, affecting homeostasis and survival of neuronal cell bodies (Cheng et al. 2010; Bridi et al. 2018), a tentative mechanism of oligomeric α-synuclein-mediated aberrant calcium dyshomeostasis promoting early neuronal pathophysiological change in LBD-vulnerable neurons, is proposed in Fig. 3.

Immunohistochemical studies reveal that among central neurons, the expression level of CaBP1 is lowest in SNc neurons, which are the most fragile in PD (Laube et al. 2002; Kim et al. 2014). The scarcity of CaBP1 in SNc neurons suggests that the smallest amount of α-synuclein oligomers can induce the aberrant CICR described here, and that this may account for by far the highest vulnerability of SNc neurons. The L-VDCC blocker, isradipine, suppresses Ca^2+^ influx via CaV1.3 L-VDCC and inhibits degeneration of SNc neurons (Chan et al. 2007), although this drug failed to slow the progression of PD (The Parkinson Study Group STEADY-PD III Investigators 2020). Given that L-VDCC blockers suppress aberrant CICR from IP_3_Rs (Yamamoto et al. 2019), and lower the risk of PD (Ritz et al. 2010; Ascherio and Schwarzschild 2016), they may reduce the risk of aberrant CICR propagation along the ER in the somatodendritic area, and may have potential in protecting LBD-vulnerable neurons from damage and the spread of Lewy body pathology.
